# Leptin receptor Gln223Arg polymorphism and breast cancer risk in Nigerian women: A case control study

**DOI:** 10.1186/1471-2407-8-338

**Published:** 2008-11-18

**Authors:** Michael N Okobia, Clareann H Bunker, Seymour J Garte, Joseph M Zmuda, Emmanuel R Ezeome, Stanley N Anyanwu, Emmanuel E Uche, Lewis H Kuller, Robert E Ferrell, Emanuela Taioli

**Affiliations:** 1 Department of Epidemiology, Graduate School of Public Health, University of Pittsburgh, Pittsburgh, PA 15261, USA; 2 Division of Cancer Prevention and Population Science, University of Pittsburgh Cancer Institute, Pittsburgh, PA 15232, USA; 3 Department of Environmental and Occupational Health, Graduate School of Public Health, University of Pittsburgh, Pittsburgh, PA 15261, USA; 4 Cancer Institute, University of Pittsburgh, Pittsburgh, PA 15261, USA; 5 Department of Human Genetics, Graduate School of Public Health, University of Pittsburgh, Pittsburgh, PA 15261, USA; 6 Department of Surgery, University of Benin Teaching Hospital, Benin City, Nigeria; 7 Department of Surgery, University of Nigeria Teaching Hospital, Enugu, Nigeria; 8 Department of Surgery, Nnamdi Azikiwe University Teaching Hospital, Nnewi, Nigeria; 9 Department of Surgery, University of Port Harcourt Teaching Hospital, Port Harcourt, Nigeria

## Abstract

**Background:**

Leptin, a 16 kDa polypeptide hormone, implicated in various physiological processes, exerts its action through the leptin receptor, a member of the class I cytokine receptor family. Both leptin and leptin receptor have recently been implicated in processes leading to breast cancer initiation and progression in animal models and humans. An A to G transition mutation in codon 223 in exon 6 of the leptin receptor gene, resulting in glutamine to arginine substitution (Gln223Arg), lies within the first of two putative leptin-binding regions and may be associated with impaired signaling capacity of the leptin receptor. This study was designed to assess the role of this polymorphism in breast cancer susceptibility in Nigerian women.

**Methods:**

We utilized a polymerase chain reaction (PCR)-based restriction fragment length polymorphism (RFLP) assay to evaluate the association between the Gln223Arg polymorphism of the leptin receptor gene and breast risk in Nigeria in a case control study involving 209 women with breast cancer and 209 controls without the disease. Study participants were recruited from surgical outpatient clinics and surgical wards of four University Teaching Hospitals located in Midwestern and southeastern Nigeria between September 2002 and April 2004.

**Results:**

Premenopausal women carrying at least one *LEPR *223Arg allele were at a modestly increased risk of breast cancer after adjusting for confounders (OR = 1.8, 95% confidence interval [CI] 1.0–3.2, p = 0.07). There was no association with postmenopausal breast cancer risk (OR = 0.9, 95% CI 0.4–1.8, p = 0.68).

**Conclusion:**

Our results suggest that the *LEPR *Gln223Arg polymorphism in the extracellular domain of the *LEPR *receptor gene is associated with a modestly increased risk of premenopausal breast cancer in Nigerian women.

## Background

Leptin, a 16 kDa polypeptide hormone produced predominantly by white adipose tissue [1], plays an important role in body weight homeostasis through effects on food intake and energy expenditure [2,3]. In addition to the regulation of body weight, leptin also influences hematopoiesis, reproduction, angiogenesis, and immune processes [4-6]. Leptin exerts its physiological action through the leptin receptor (*LEPR*), a member of the class I cytokine receptor family. The leptin receptor is a single transmembrane protein and has several alternatively spliced isoforms (one long isoform and several short isoforms) that are distributed in many tissues [7,8]. *LEPRs *have been detected in human breast epithelial cell lines, breast cancer-derived cell lines (T47-D and MCF-7) and in human breast cancer tissue specimens [9-11]. In addition, leptin consistently stimulates the proliferation of benign and malignant epithelial breast cells in vitro as measured by DNA synthesis, cellular density and up-regulation of downstream regulators of cellular proliferation [9-12].

Several single nucleotide polymorphisms (SNPs) in the *LEPR *gene have been described. Chung et al. [13] identified three allelic variants associated with amino acid changes (Lys109Arg, Gln223Arg, and Lys656Asn), three silent mutations (nt 1222 T→C, nt 3217 A→G, and nt 3250 G→A), and four intronic sequence variants. All three amino acids (Lys109Arg, Gln223Arg, and Lys656Asn) are conserved among rat, mouse, and human species [14,15], but only Gln223Arg and Lys656Asn result in changes in charge (neutral to positive and positive to neutral, respectively) and are, therefore, most likely to have functional consequences. The Gln223Arg polymorphism is within the region encoding the extracellular domain of the leptin receptor and therefore, the amino acid change affects all forms of the receptor. It has been shown that the *LEPR *Gln223Arg polymorphism is associated with variation in ligand binding; higher levels of ligand binding activity has been demonstrated in individuals homozygous for the G (*LEPR *Arg223Arg) allele than for carriers of the A (*LEPR *223Gln) allele [16].

Despite these premises, very few studies have considered the role of serum leptin levels and polymorphisms in leptin and *LEPR *genes in breast cancer risk [17-23]. Four studies [17-20] on plasma leptin levels and breast cancer risk have shown conflicting results; one in the Italian population [17] and another among the Chinese [18] reported significant increased risk of breast cancer with higher serum leptin levels while two other studies [19,20] in the Greek and U.S. populations, respectively, found no associations. Of two studies that evaluated relationship between *LEPR *Gln223Arg polymorphism and serum leptin levels, one study among college students in Greece [21] reported significantly higher leptin levels in heterozygote (*LEPR *Gln223Arg) and homozygote (*LEPR *Arg223Arg) carriers of the G allele; the second study by Quinton et al. [16] among postmenopausal British women reported lower leptin levels among carriers of the G allele. Only two studies [22,23] have examined allelic variants of the *LEPR *genes in breast cancer susceptibility; one [22] reported positive association among Tunisian women while the other [23] found no association between these variants and breast cancer risk in Korean women. We are unaware of any study of this susceptibility marker and breast cancer risk in black populations. Although, several polymorphisms have been described in the *LEPR *gene, we have chosen in this exploratory study to evaluate the role of the *LEPR *Gln223Arg polymorphism in breast cancer susceptibility in Nigerian women since it is the most studied functional *LEPR *gene polymorphism in human subjects.

## Methods

### Subjects

The recruitment of participants for this study from four University Teaching Hospitals located in southern Nigeria (University of Benin Teaching Hospital, Benin City; Nnamdi Azikiwe University Teaching Hospital, Nnewi; University of Nigeria Teaching Hospital, Enugu; and University of Port Harcourt Teaching Hospital, Port Harcourt between September 2002 and April 2004) has been described elsewhere [24,25]. Briefly, 209 women with confirmed breast cancer and 209 age-matched control subjects without the disease or any other malignancy were recruited during surgical outpatient visits or hospital admissions. Control subjects were being treated in the same hospitals for non-malignant diseases including unintentional injuries, acute inflammatory diseases such as chronic duodenal ulcer, appendicitis, pelvic inflammatory disease and urolithiasis. The study protocol was approved by the Institutional Review Board of University of Pittsburgh and the Ethics and Research Committees of the Nigerian institutions prior to commencement. Women with suspected breast cancer without histological confirmation and those that refused sample donation were excluded from the study.

Study participants signed informed consent after detailed explanation of the purpose, risks and benefits, confidentiality and rights of participants prior to recruitment. Data in respect of reproductive history, demographic characteristics, occupational exposure to pesticides and fertilizers and lifestyle choices including alcohol consumption and cigarette smoking were obtained using interviewer-administered questionnaires. Anthropometric measurements including height, weight, waist and hip circumferences were taken at the end of the interview.

### Sample Donation and Preparation

Ten milliliters (ml) blood sample was collected into one 10 ml K_3_-EDTA vacutainer tube from each of the study participants at the end of the interview. The buffy coat was prepared by centrifuging whole blood 2500 × g for 10 min at room temperature. All the samples were stored at -20°C in the Nigerian coordinating center at the University of Benin Teaching Hospital and later shipped to University of Pittsburgh in dry ice. Samples were stored at -80°C at the University of Pittsburgh until DNA extraction. DNA extraction from buffy coats (and clots for subjects with no buffy coats) was carried out using QIAamp DNA Mini Kits (for buffy coats) and QIAamp DNA Midi Kits (for blood clots) protocols (QIAGEN Inc, Valencia, CA). The extracted DNA was stored at 4°C until used for polymerase chain reaction (PCR) and restriction fragment length (RFLP) analysis.

### PCR and RFLP analysis

Genomic DNA from the cases and control subjects were analyzed for the presence of the A to G mutation at codon 223 of the *LEPR *gene by a PCR-based restriction fragment length polymorphism (RFLP) assay. PCR amplification of an 80 bp fragment of the *LEPR *gene, including part of exon 6 that contains the polymorphism was carried out using forward primer: AAACTCAACGACACTCTCCTT and reverse primer: TGAACTGACATTAGAGGTGAC. A 50 μl PCR reaction mixture containing 2 μl of genomic DNA, 5 μl of deoxynucleotide triphosphates, 3 μl each of forward and reverse primers, 5 μl of 10× buffer, 1.5 μl of MgCl_2 _and 0.5 μl of Taq polymerase was placed in a thermocycler. After denaturing for 5 min at 95°C, the DNA was amplified for 35 cycles at 95°C for 30 sec, 59°C for 45 sec, and 72°C for 60 sec, followed by a 5 min extension at 72°C. A positive control containing genomic DNA and a negative control containing everything except DNA were included in the PCR experiment. Five μl of each PCR product, including the controls, were verified on a 3% agarose gel to ensure that the expected 80 bp product was generated.

Restriction digest for the DNA fragment was carried out using Msp 1 restriction enzyme. Fifteen μl of the PCR product was digested for 16 h overnight at 37°C with 10 units of Msp 1 (New England Biolabs). The product of the restriction digest was mixed with 10 μl of loading dye and verified on a 3% agarose gel (with Ethidium bromide) electrophoresis in a 1× Tris-Borate-EDTA buffer at 200 V for 60 min. The presence of an A at position 668 (*LEPR *codon 223) generated a unique 80 bp fragment, while the 80 bp fragment was divided into unique 58 bp and 22 bp fragments when position 668 contains a G. The gels were visualized by UV light and the RFLP gel electrophoresis products were read by two independent persons who were unaware of the identities of samples as either cases or controls. DNA extraction and RFLP assays employing Msp 1 restriction enzyme were successful in 209 cases and 209 control subjects; therefore the analysis is restricted to this sample of women.

### Statistical Analyses

Statistical analysis was carried out using the Statistical Analysis System (SAS) software (Version 8.0). Although the original data was matched by age, the analysis of the genetic data was conducted on the unmatched sample, since DNA was not available on all the study participants. Therefore an unmatched analysis was conducted and an adjustment for age was performed. Unconditional logistic regression was used to assess the association between the *LEPR *Gln223Arg genotypes and breast cancer risk in the whole sample. Stratified analyses according to menopausal status were then carried out. Relevant risk factors that were identified as significant predictors of breast cancer risk were controlled for in the multivariate logistic regression models.

## Results

### Demographic Characteristics

A total of 418 female participants were recruited from the surgical outpatient clinics and surgical wards of the four University Teaching Hospitals in southern Nigeria. Two hundred and nine of them were being managed for various stages of breast cancer while the remaining 209 women, being treated for various non-malignant diseases in the same institutions served as control subjects. The ages of the cases and control subjects were similar with mean ages of 46.1 ± 12.63 years and 47.1 ± 13.50 years for cases and controls, respectively. Details on the associations between anthropometric, demographic and reproductive variables are reported elsewhere [26,27].

### Allele and Genotype Frequencies in All women

Our results indicate that the *LEPR *Gln223Arg variant is highly polymorphic in the Nigerian population. The distribution of the *LEPR *Gln223Arg alleles and genotypes are shown in Table 1. When all study participants were considered together, the distribution of the *LEPR *223Gln allele was similar in cases and controls with allele frequencies of 0.49 and 0.51, respectively while the frequency of the *LEPR *223Arg allele was 0.51 and 0.49, respectively. The homozygous *LEPR *Gln223Gln genotype was slightly less common in the cases compared to the controls with genotype frequencies of 0.22 and 0.27, respectively while the heterozygous *LEPR *Gln223Arg genotype was equally distributed among cases and controls with frequency of 0.51 in both cases and control subjects. The homozygous Arg223Arg genotype was slightly more common in the cases compared to the controls with frequencies of 0.27 and 0.22, respectively. The distribution of the *LEPR *223Gln and 223Arg alleles among the controls were in Hardy-Weinberg equilibrium overall and in both premenopausal and postmenopausal women.

**Table 1:** Distribution of Leptin receptor (*LEPR*) alleles and genotypes in relation to breast cancer risk (unconditional logistic regression)

	**Cases**	**Controls**	**OR (95% CI)**	**P-value**	OR (95% CI)*	**P-value**
**All women**	(n = 209)	(n = 209)				
**Allele frequencies**						
*LEPR *(Gln)	0.49	0.51				
*LEPR *(Arg)	0.51	0.49				
						
**Genotype frequencies**						
*LEPR *(Gln/Gln)	46 (22.0)	56 (26.8)	1.0			
*LEPR *(Gln/Arg)	107 (51.2)	107 (51.2)	1.2 (0.8–2.0)	0.42	1.2 (0.8–2.0)	0.40
						
*LEPR *(Arg/Arg)	56 (26.8)	46 (22.0)	1.5 (0.8–2.6)	0.16	1.6 (0.9–3.2.8)	0.13
*LEPR *(Gln/Gln	46 (22.0)	56 (26.8)	1.00			
*LEPR *(Gln/Arg) + (Arg/Arg)	163 (78.0)	153 (73.2)	1.3 (0.8–2.0)	0.26	1.3 (0.8–2.1)	0.23
						
**Pre-menopausal women**	(n = 114)	(n = 121)				
Allele frequencies						
*LEPR *(Gln)	0.46	0.54				
*LEPR *(Arg)	0.54	0.46				
						
**Genotype frequencies**						
*LEPR *(Gln/Gln)	23 (20.2)	38 (31.4)	1.00			
*LEPR *(Gln/Arg)	60 (52.6)	54 (44.6)	1.8 (1.0–3.5)	0.06	1.8 (0.9–3.3)	0.07
*LEPR *(Arg/Arg)	31 (27.2)	29 (24.0)	1.8 (0.9–3.6)	0.12	1.8 (0.8–3.7)	0.14
						
*LEPR *(Gln/Gln	23 (20.2)	38 (31.4)	1.00			
*LEPR *(Gln/Arg) + (Arg/Arg)	91 (79.8)	83 (68.6)	1.8 (1.0–3.3)	0.05	1.8 (1.0–3.2)	0.07
						
**Postmenopausal women**	(n = 95)	(n = 88)				
**Allele frequencies**						
*LEPR *(Gln)	0.49	0.51				
*LEPR *(Arg)	0.51	0.49				
						
**Genotype frequencies**						
*LEPR *(Gln/Gln)	23 (24.2)	18 (20.5)	1.00			
*LEPR *(Gln/Arg)	47 (49.5)	53 (60.2)	0.7 (0.3–1.4)	0.33	0.7 (0.4–1.5)	0.42
*LEPR *(Arg/Arg)	25 (26.3)	17 (19.3)	1.2 (0.5–2.8)	0.75	1.2 (0.5–3.0)	0.62
						
*LEPR *(Gln/Gln	23 (24.2)	18 (20.5)	1.00			
*LEPR *(Gln/Arg) + (Arg/Arg)	72 (75.8)	70 (79.5)	0.8 (0.4–1.6)	0.54	0.9 (0.4–1.8)	0.68

*Adjusted for age and waist/hip ratio (WHR)

### Premenopausal Women

The DNA extraction and RFLP Msp 1 assays were successful in 114 premenopausal breast cancer cases and 121 control subjects. The *LEPR *223Gln allele was less frequent in premenopausal breast cancer cases compared to those without breast cancer with allele frequencies of 0.46 and 0.54, respectively. The wild-type *LEPR *Gln223Gln genotype was less frequent in the women with breast cancer compared to those without the disease, with genotype frequencies of 0.20 and 0.31, respectively. (Table 1).

### Postmenopausal Women

There was no apparent difference in the distribution of the *LEPR *223Gln and *LEPR *223Arg alleles in postmenopausal women with breast cancer compared to the control subjects. The *LEPR *Gln223Gln genotype frequencies were 0.24 in cases and 0.21 in the controls as shown in Table 1.

### LEPR Gln223Arg Genotypes and Breast Cancer Risk in All Women

Compared to women with the *LEPR *Gln223Gln genotype, those harboring the heterozygous *LEPR *Gln223Arg genotype (OR = 1.2, 95% CI 0.8–2.0, p = 0.42) and the homozygous mutant *LEPR *Arg223Arg genotype (OR = 1.5, 95% CI 0.8–2.6, p = 0.16) had no significant increased risk of breast cancer.

The presence of at least one *LEPR *223Arg allele (*LEPR *Gln223Arg + *LEPR *Arg223Arg genotypes) was not associated a significant risk of breast cancer in these women (OR = 1.3, 95% CI 0.8–2.0, p = 0.26). Adjusting for waist/hip ratio, a surrogate measure of obesity did not significantly alter the risk profile in these women as shown in Table 1.

### Premenopausal Women

Among premenopausal women, there was a marginally increased risk of breast cancer with the *LEPR *223Arg allele (*LEPR *Gln223Arg + *LEPR *Arg223Arg): OR = 1.8, 95% CI 1.0–3.3, p = 0.05 (Table 1). The heterozygous *LEPR *Gln223Arg genotype was associated with a modestly increased risk of breast cancer (OR = 1.8, 95% CI 1.0–3.5, p = 0.06). The homozygous *LEPR *Arg223Arg genotype was also associated with some modest increase in breast cancer risk (OR = 1.8, 95% CI 0.9–3.6, p = 0.12). These risk profiles were unaltered when the analysis was adjusted for waist/hip ratio and age (*LEPR *Gln223Arg + *LEPR *Arg223Arg OR = 1.8, 95% CI 1–3.2, p = 0.07; *LEPR *Gln223Arg genotype OR = 1.8, 95% CI 0.9–3.3, p = 0.07 and *LEPR *Arg223Arg genotype OR = 1.8, 95% CI 0.8–3.7, p = 0.14).

### Postmenopausal Women

Among postmenopausal women there was no significant relationship between the *LEPR *Gln223Arg polymorphism and breast cancer risk (Table 1). Adjusting for waist/hip ratio did not significantly alter the risk profiles.

## Discussion

The results of our study showed that the *LEPR *Gln223Arg variant is highly polymorphic in the Nigerian population. The *LEPR *223Arg allele frequency of 0.49 in our study participants is higher than figures reported in Caucasians (0.45), Pima Indians (0.32), Arabs (0.34) but much lower than figures in Asian populations (0.85) [28]. Although a study of mixed U.S. population by Chung et al. [13] reported little difference in *LEPR *223Arg allele frequency by racial groups, the study recruited only 26 blacks and racial admixture in the U.S. population may partly account for their finding. The International Hapmap Project reported *LEPR *223Arg allele frequencies of 0.61 among Yorubas in Ibadan, Nigeria and 0.53 in African Americans. [29]

The evaluation of the relationship of the *LEPR *Gln223Arg polymorphism and breast cancer risk showed that premenopausal women carrying at least one *LEPR *223Arg allele were at a modestly increased risk of breast cancer (OR = 1.8, 95% CI 1.0–3.3, p = 0.05); the risk was unaltered after adjusting for waist/hip ratio and age (OR = 1.8, 95% CI 1.0–3.2, p = 0.07). There was no association between the *LEPR *Gln223Arg polymorphism and breast cancer risk in postmenopausal women. Two studies [22,23] evaluated the *LEPR *Gln223Arg polymorphism in relation to breast cancer risk; one [22] in the Tunisia population reported significantly increased risk in both premenopausal and postmenopausal women in a dose dependent manner (OR = 1.68, 95% CI 1.12–2.50 and OR = 2.26, 95% CI 1.31–3.90 for the *LEPR *Gln223Arg and Arg223Arg genotypes, respectively). In addition, the authors noted that the presence of the *LEPR *223Arg allele was associated with poorer overall survival. The second study by Woo et al. [23] found no association between the polymorphism and breast cancer risk in both premenopausal and postmenopausal Korean women (OR = 0.54, 95% CI 0.19–1.81). The authors noted the rarity of the *LEPR *223Gln allele in the Korean population (allele frequency, 0.09) and the small sample size of their study (45 breast cancer cases and 45 control subjects). Some investigators [16,18,21] have examined the relationship between variants of the *LEPR *Gln223Arg polymorphism and serum levels of leptin since there is some evidence associating serum leptin levels with breast cancer risk [17,18]. In a study involving 118 college students in Greece (62 females and 56 males), Yiannkouris et al. [21] reported significantly higher serum leptin levels in individuals who were homozygous *LEPR *Arg223Arg compared with those harboring at least one *LEPR *223Gln allele. Another study of 220 postmenopausal women in the U.K. found significant association between serum leptin levels and the *LEPR *genotype [16].

Although the actual mechanisms of leptin's role in breast cancer risk are not completely known, several lines of evidence provide support for leptin's broader physiological role, including the regulation of several neuroendocrine axes, some of which play a significant role in the pathogenesis of breast cancer. Leptin treatment corrects the hypogonadism of leptin-deficient ob/ob mice [30,31] and starved normal mice [32], accelerates the onset of puberty in rodents and increasing leptin levels may signal the onset of puberty in boys and girls [33,34]. In addition, leptin's pulsatile secretion is synchronized with the pulsatility of luteinizing hormone and estradiol in normal women [35]. Leptin has been shown to regulate GH secretion [36] and serum leptin levels have been associated with circulating IGF-1 and IGFBP-3 levels in normal and GH-deficient humans [34,37]. In addition, circulating leptin levels have been associated with certain life-style factors, such as smoking and alcohol intake [34]. Interestingly, the above endocrine axes and life-style factors have also been implicated in the pathogenesis of breast cancer via leptin's interaction with IGF-I and IGFBP-3 [38,39].

Tumor markers that are elevated in breast cancer can upregulate leptin production. These factors, including TNF-α, IL-1α, IL-1β, VEGF, and fibroblast growth factor 2 (FGF-2), raise leptin levels and promote tumor growth and differentiation [40,41]. In experimental studies, leptin consistently stimulated human breast epithelial cell lines and breast cancer-derived cell lines resulting in increased DNA synthesis evaluated by thymidine incorporation test and increased cellular growth estimated by cellular density [9-12]. Several down-stream effects of leptin signaling leading to cellular proliferation were also observed including increased expression of phosphorylated signal transducers (STAT3), extracellular signal-regulated kinase (ERK), mitogen-activated protein kinases (MAPK) and transcript activator protein (AP-1), and also increased expression of cyclin dependent kinase-2 and cyclin D1, two cell-cycle regulating proteins [9,10]. It has been suggested that the single amino acid change in the *LEPR *gene (*LEPR *Gln223Arg), a glutamine for an arginine with a change from neutral to positive, could affect the functionality of the receptor and alter its signaling capacity [13,42,43]. The finding of higher leptin binding activity (LBA) levels in homozygous carriers of the G allele (*LEPR *Arg223Arg) and higher levels of leptin in the *LEPR *Arg223Arg homozygotes and our finding of increased premenopausal breast cancer risk in women carrying the *LEPR *223Arg allele provides supportive evidence for this proposition. It is possible that the leptin polymorphism is in linkage disequilibrium with other genes that play a role in breast cancer risk.

An important observation is the paucity of epidemiological literature on the role of polymorphisms in the leptin receptor (*LEPR*) gene in breast cancer susceptibility in most populations and its complete absence in sub-Saharan African populations. Much has been said about the mechanisms of leptin-induced carcinogenesis in animal models and correlation of serum leptin levels with breast cancer risk [17-20]. The effect of genotypes such as *LEPR *Gln223Arg polymorphism on breast cancer may vary from one population to the other as a result of marked differences in the distribution of the alleles in different populations. The finding of interaction between *LEPR *Gln223Arg genotypes and menopausal status and obesity may also partly explain the differences in reports from various populations. In the developed countries with a much higher percentage of postmenopausal breast cancer and a higher proportion of obese women, *LEPR *Gln223Arg polymorphism may be expected to have more impact on risk of the disease compared to the Nigerian population with a lower prevalence of obesity. Some of the limitations of this study include use of both incident and prevalent cases of breast cancer. Given the report of poorer prognosis associated with the *LEPR *223Arg allele in some studies, it is possible that some patients harboring this allele might have died earlier leaving us with more patients with the *LEPR *223Gln allele. However, such a bias if prevent would result in underestimation of the risk associated with the putative high risk *LEPR *223Arg allele. There is currently no breast cancer screening program in Nigeria; therefore patients with early pre-clinical disease may have been missed in our study. Use of hospital controls is necessitated by the limited research infrastructure such as poor communication facilities and lack of population-based cancer registries in developing countries such as Nigeria.

Another issue of concern is the sample size of this study. In fact, we estimate that with an allele frequency of .49 in all women combined, a sample size of 139 cases and controls provides a power of 80% to detect an odds ratio (OR) of 2.0. There were a total of 209 cases and 209 controls in our study population indicating that this sample size has adequate statistical power to detect an OR of 2.0. However, when we stratify by menopausal status, the number of cases and controls become much lower, thus decreasing the statistical power of the study. For example, a sample size of 137 premenopausal cases and 137 premenopausal controls would be adequate to detect an odds ratio of 2.0 while there were 114 premenopausal cases and 121 premenopausal controls in our study. It is possible that variations in LEPR polymorphisms and other low penetrance genes may contribute to the marked worldwide variation in breast cancer incidence, with the highest age-standardized incidence rate (ASR) in North America (ASR, 99.4 per 100,000) and the lowest age-standardized incidence rates in sub-Saharan Africa (ASR, 27.8 per 100,000 in West Africa, 19.5 per 100,000 in East Africa and 16.5 per 100,000 in Central Africa) [44].

## Conclusion

In conclusion, this study has demonstrated a modestly increased risk of premenopausal breast cancer in women harboring the *LEPR *223Arg allele of the *LEPR *Gln223Arg polymorphism of the leptin receptor gene. To the best of our knowledge, ours is the first study to provide information on the role of *LEPR *Gln223Arg polymorphism in breast cancer risk in sub-Saharan African women, a population characterized by paucity of epidemiological literature on the determinants of breast cancer and other malignancies.

## Abbreviations

*LEPR*: Leptin receptor; DNA: Deoxyribonucleic acid; PCR: Polymerase chain reaction; SNP: Single Nucleotide Polymorphism; RFLP: Restriction fragment length polymorphism; STAT3: Signal transducers of activation of transcription; ERK: Extracellular signal-regulated kinase; MAPK: Mitogen-activated protein kinase; SAS: Statistical analysis systems

## Competing interests

The authors declare that they have no competing interests.

## Authors' contributions

MNO, CHB, ET, SJG, REF, LHK, participated in conceptualization, design of the study and preparation of manuscript. MNO, ERE, SNA, and EEU recruited study participants from Nigeria and organized the transfer of biological samples to the University of Pittsburgh. MNO, CHB, ET, SJG and JMZ carried out the genetic analysis.

## Pre-publication history

The pre-publication history for this paper can be accessed here:


